# MRI-based radiomics model for preoperative prediction of 5-year survival in patients with hepatocellular carcinoma

**DOI:** 10.1038/s41416-019-0706-0

**Published:** 2020-01-15

**Authors:** Xiao-Hang Wang, Liu-Hua Long, Yong Cui, Angela Y. Jia, Xiang-Gao Zhu, Hong-Zhi Wang, Zhi Wang, Chong-Ming Zhan, Zhao-Hai Wang, Wei-Hu Wang

**Affiliations:** 1 Key Laboratory of Carcinogenesis and Translational Research (Ministry of Education/Beijing), Department of Radiation Oncology, Peking University Cancer Hospital and Institute, Beijing, China; 2 Key Laboratory of Carcinogenesis and Translational Research (Ministry of Education/Beijing), Department of Radiology, Peking University Cancer Hospital and Institute, Beijing, China; 3 Department of Radiation Oncology and Molecular Radiation Sciences, Johns Hopkins University School of Medicine, Baltimore, MD USA; 4 Blot Info & Tech (Beijing) Co. Ltd, Beijing, China; 5 Department of Hepatobiliary Surgery, The Fifth Medical Center of Chinese PLA General Hospital, Beijing Institute of Infectious Diseases, Beijing, China

**Keywords:** Cancer models, Nomograms, Translational research, Cancer imaging

## Abstract

**Background:**

Recurrence is the major cause of mortality in patients with resected HCC. However, without a standard approach to evaluate prognosis, it is difficult to select candidates for additional therapy.

**Methods:**

A total of 201 patients with HCC who were followed up for at least 5 years after curative hepatectomy were enrolled in this retrospective, multicentre study. A total of 3144 radiomics features were extracted from preoperative MRI. The random forest method was used for radiomics signature building, and five-fold cross-validation was applied. A radiomics model incorporating the radiomics signature and clinical risk factors was developed.

**Results:**

Patients were divided into survivor (*n* = 97) and non-survivor (*n* = 104) groups based on the 5-year survival after surgery. The 30 most survival-related radiomics features were selected for the radiomics signature. Preoperative AFP and AST were integrated into the model as independent clinical risk factors. The model demonstrated good calibration and satisfactory discrimination, with a mean AUC of 0.9804 and 0.7578 in the training and validation sets, respectively.

**Conclusions:**

This radiomics model is a valid method to predict 5-year survival in patients with HCC and may be used to identify patients for clinical trials of perioperative therapies and for additional surveillance.

## Background

Primary liver cancer (PLC) is the third leading cause of cancer-related death worldwide, with an estimated 5-year overall survival rate of 18%.^1^ Hepatocellular carcinoma (HCC) is the most common pathological type of PLC, accounting for 70–85% of cases.^2^ Hepatectomy is the primary curative treatment for patients with early-stage HCC with well-preserved liver function.^3^ However, tumour recurrence remains the major cause of death after surgery, with the postoperative 5-year recurrence rate approaching 70%.^4^ In patients at high-risk for recurrence, adjuvant therapy may be warranted, despite the current lack of proven effective treatment.^5^ Some retrospective and prospective studies have suggested that adjuvant transarterial chemoembolisation could delay recurrence and improve survival in high-risk patients with HCC.^6,7^ Retrospective studies from our own group indicated that postoperative intensity-modulated radiotherapy may be a favourable option in patients with HCC with narrow-margin resection or microscopic vascular invasion.^8,9^ Prognosis classification is essential for individualised treatment; however, no generally accepted approach for risk stratification in HCC is currently available. Therefore, the need remains for a feasible and reproducible method to identify high-risk patients with HCC.

Several clinicopathologic characteristics and gene expression parameters have been proved to help predict biological aggressiveness and clinical prognosis in HCC.^10,11^ However, most of the parameters are based on postoperative pathologic examination, which may be susceptible to observer variability and fail to aid in preoperative decision making.

In recent years, with the rapid development of artificial intelligence, data-mining technology has made breakthroughs in medical imaging analysis, giving rise to the new field of radiomics. This research approach utilises high-throughput extraction of feature data from radiographic images,^12^ and can potentially develop models to predict lesion phenotypes and prognosis in a non-invasive manner.^13,14^

To our knowledge, there are relatively limited radiomics analysis data about prognosis estimation in HCC, with most of the radiomics models established on the basis of computed tomography (CT) images,^15–19^ and only a few studies investigating the role of magnetic resonance imaging (MRI),^20–23^ especially contrast-enhanced MRI. Moreover, previous MRI-based radiomics analyses of patients with HCC were based on a few MRI sequences from small-sample studies, and no MRI-based radiomics model for long-term survival prediction in HCC is currently available. Therefore, the purpose of this study was to develop a radiomics model based on four conventional MRI sequences to predict 5-year survival in patients with HCC in the preoperative setting.

## Methods

### Patients

**Fig. 1:**
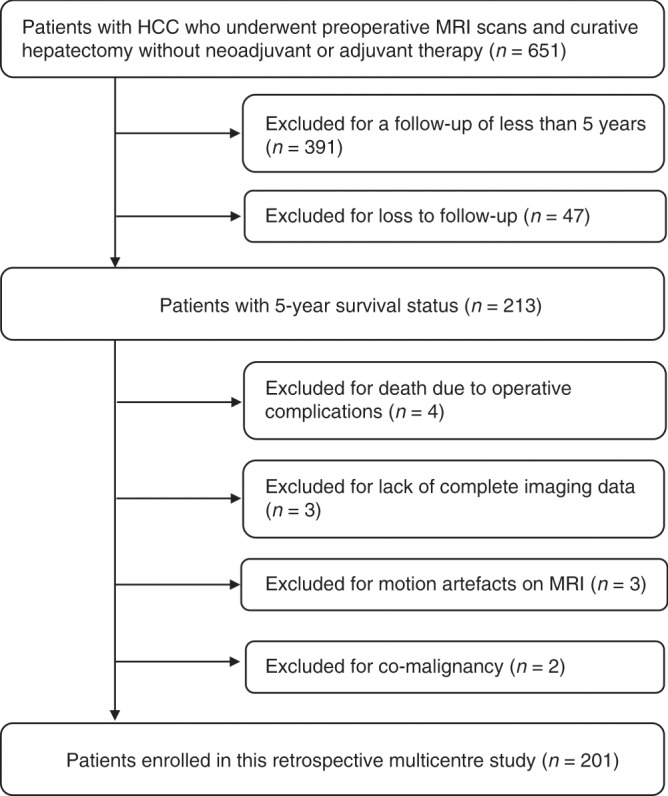
Study recruitment process. HCC hepatocellular carcinoma, MRI magnetic resonance imaging.

This retrospective, multicentre study was approved by the institutional review board, and the requirement for individual informed consent was waived because of the retrospective nature of the study. This study was performed at two medical centres: Peking University Cancer Hospital and The Fifth Medical Center of Chinese PLA General Hospital. From August 2010 to September 2016, consecutive patients with surgically resected, pathologically confirmed HCC were screened. The inclusion criteria were as follows: (1) patients with curative hepatectomy, defined as complete removal of all macroscopic tumours with negative resection margins, (2) no lymph node or extrahepatic metastasis, (3) no major vascular invasion, (4) patients who underwent preoperative contrast-enhanced MRI, (5) no neoadjuvant or adjuvant therapy and (6) postoperative follow-up for at least 5 years (unless death occurred). The exclusion criteria were as follows: (1) patients with co-malignancy, (2) death due to operative complications, (3) lack of complete imaging data, including T1-weighted imaging (T1WI), T2-weighted imaging (T2WI), diffusion-weighted imaging (DWI) and dynamic contrast-enhanced imaging (DCEI) and (4) motion artefacts on MRI. The final study population included 201 patients. The study recruitment process is shown in Fig. 1.

Preoperative clinical characteristics, including age, sex, hepatitis B surface antigen (HBsAg) and hepatitis C Virus antibody (HCV-ab) status, cirrhosis, alpha-fetoprotein (AFP), alanine aminotransferase (ALT), aspartate aminotransferase (AST), total bilirubin (TB), direct bilirubin (DB), albumin (ALB), platelet count (PLT), prothrombin time (PT), international normalised ratio (INR), Child–Pugh class, MRI-reported tumour number, MRI-reported tumour size and Barcelona Clinic Liver Cancer (BCLC) stage, were derived from electronic medical records. Laboratory examination results were obtained from blood tests within 2 weeks before surgery. In accordance with the normal range at our institutions, the threshold levels for abnormal AFP, ALT, AST, TB, DB, ALB, PLT, PT and INR were 7 ng/mL, 40 U/L, 45 U/L, 20 μmol/L, 6 μmol/L, 35 g/L, 100 × 10^9^/L, 14 s and 1.5, respectively.

### Follow-up

All patients were consistently followed up every 3 to 6 months after surgery based on the AFP level and imaging examinations. Tumour recurrence was determined based on radiologic evidence of intra- or extrahepatic new tumours. Five-year survival status was defined as survival or death at 5 years after surgery.

### MRI

MRI scans were obtained within 2 weeks before surgery. MRI was performed using a 3.0-T scanner (Discovery MR 750, GE Healthcare, Milwaukee, Wisconsin, USA) with a 32-channel phased-array body coil, with patients in the supine position with breath holding. T1WI was obtained using gradient echo with a repetition time (TR) of 3.5–4.0 ms, echo time (TE) of 1.5–2.0 ms, field of view (FOV) of 300 × 400 mm, matrix of 256 × 256, flip angle of 10°, section thickness of 6 to 7 mm and intersection gap of 1 to 2 mm. T2WI was obtained using spin echo with a TR of 2500 ms, TE of 90 ms, FOV of 300 × 400 mm, matrix of 384 × 256, flip angle of 20°, section thickness of 6–7 mm, and intersection gap of 1–2 mm. DWI was acquired with 2 b-values (0 and 800 s/mm^2^), TR of 2600 ms, TE of 59.5 ms, FOV of 300 × 400 mm, matrix of 128 × 128, flip angle of 90°, section thickness of 6–8 mm and intersection gap of 1–2 mm. T1-weighted DCEI was acquired using 0.1 mmol/kg gadolinium-diethylenetriamine pentaacetic acid (Gd-DTPA) at a rate of 2.5 mL/s in the arterial (a scanning delay of 20–25 s), portal venous (a scanning delay of 60–70 s) and equilibrium (a scanning delay of 180 s) phases.

### Statistical analysis

Categorical variables were expressed as number and percentage and were compared using the *X*^2^ or Fisher’s exact test. Uni- and multivariable logistic regression analyses were performed to identify independent clinical risk factors associated with 5-year survival. The candidate factors for univariable analysis were age, sex, HBsAg/HCV-ab status, cirrhosis, AFP, ALT, AST, TB, DB, ALB, PLT, PT, INR, Child–Pugh class, MRI-reported tumour number, MRI-reported tumour size and BCLC stage. Odds ratio and 95% confidence interval (CI) were calculated. The variables with a *P-*value < 0.10 in the univariable analysis were selected as candidates for the multivariable analysis. A two-tailed *P-*value < 0.05 was considered a statistically significant difference. Five-fold cross-validation was applied for robust estimation. Patients were divided into five random subgroups of approximately equal size, with each subgroup regarded as a validation set and the remaining four-fifths of patients used as the training set. The process was repeated five times with different subgroups, forming five training sets (training sets 1, 2, 3, 4 and 5) and five corresponding validation sets (validation sets 1, 2, 3, 4 and 5). The *X*^2^ test, Fisher’s exact test and logistic regression analysis were conducted using SPSS version 23.0 (IBM Corporation, Armonk, NY, USA). Region of interest (ROI) segmentation, MRI normalisation and feature extraction were performed using Precision Medicine Open Platform version 2.0.1 (https://client.blothealth.com). Feature selection and model construction were conducted using Pycharm version 2017.3.2 (https://www.jetbrains.com).

### ROI segmentation

T1WI, T2WI, DWI and DCEI were exported as Digital Imaging and Communication in Medicine (DICOM) files. The ROI, defined as all tumour regions in each axial slice, was semi-automatically segmented by a radiation oncologist (Xiang-Gao Zhu, with 5 years of experience in PLC radiotherapy), and checked by a radiology expert (Yong Cui, with 10 years of experience in PLC imaging) to minimise possible bias. Disagreements were verified by a senior expert (Wei-Hu Wang, with 20 years of experience in PLC radiotherapy).

### MRI normalisation and radiomics feature extraction

Considering that MRI examinations were performed at different centres and there was some inhomogeneity between scanners, we performed MRI signal-intensity normalisation to correct the scanner effect. The ROI was automatically extracted from T1WI, T2WI, DWI and DCEI. Three-dimensional reconstruction was performed, and ROI images were resampled to a voxel size of 1 × 1 × 1 mm, which could correct the pixel-spacing difference and restore the tumour volume. Four groups of imaging features were extracted: (1) 540 histogram of oriented gradient features, (2) 42 texture features, (3) 48 wavelet features and (4) 156 statistical features. The final set contained 786 features for each sequence, resulting in a total of 3144 features per patient. All features were calculated in three-dimensional tumour volumes.

### Feature selection and radiomics signature construction

Invalid features were removed, and normalisation was applied to the remaining features for the sake of comparison. The Gini coefficient in the random forest algorithm was used to select the most survival-related features. The Gini coefficient was defined as follows:

$${\mathrm{Gini}}\left( D \right) = 1 - \mathop {\sum }\limits_{i = 1}^n pi^2,$$

$$\Delta {\mathrm{Gini}}(X) = {\mathrm{Gini}}(D) - {\mathrm{Gini}}_X(D),$$

where *D* is the entire sample, *n* is the number of categories, *pi* (*i* = 1, 2, …, *n*) is the probability of each category, and *X* is a radiomics feature.

The features with a maximum ${\mathrm{\Delta }}{\mathrm{Gini}}\left( X \right)$ value were selected, which could be diverse in different training sets. Based on the selected features, a radiomics signature was developed using the random forest method. The appropriate value of parameters in the random forest algorithm is crucial to the performance of the radiomics signature. The number of trees in the forest was used to ensure adequate fitness, and maximum leaf node was used to inhibit overfitting.

### Development and evaluation of radiomics model

**Fig. 2:**
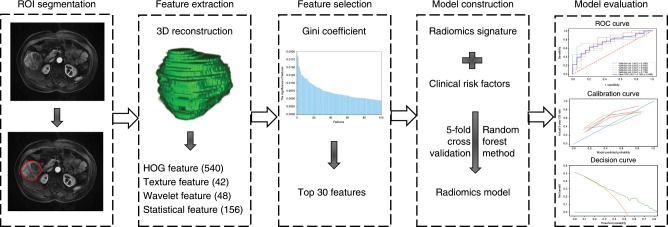
Workflow of necessary steps in this study. The region of interest (ROI) in each transverse section was semi-automatically segmented on T1-weighted, T2-weighted, diffusion-weighted and dynamic contrast-enhanced magnetic resonance images. After three-dimensional reconstruction of the ROI, 3144 features, including 786 for each sequence, were extracted, and the top 30 were selected via Gini coefficient. Based on the selected features and clinical risk factors, a radiomics model was developed using the random forest method and five-fold cross-validation. The performance of the radiomics model was evaluated according to receiver-operating characteristic, calibration and decision curves. HOG histogram of oriented gradient, ROC receiver-operating characteristic.

The individualised radiomics model incorporating the radiomics signature and independent clinical risk factors was constructed using the random forest method. Evaluation of the model included discrimination, calibration and clinical usefulness. Discrimination performance was quantified based on area under the curve (AUC) of the receiver-operating characteristic (ROC) curve. Calibration performance was assessed based on agreement between predicted and actual 5-year survival rates in the calibration curve. Clinical usefulness was estimated based on the net benefit of the model across different threshold probabilities in decision curve analysis.^24^ The study workflow is detailed in Fig. 2.

## Results

### Clinical characteristics

A total of 201 patients with HCC were analysed in this study. One hundred sixty-eight patients had hepatitis B/C virus infection, and 149 (88.7%) received antiviral therapy regularly. One hundred fifty-eight patients (78.6%) had a history of cirrhosis. The median follow-up was 52.5 months (range, 5.2–93.1 months); 97 patients survived 5 years or more and 104 died within 5 years after surgery. Sixty-eight surviving patients were free from HCC within 5 years after surgery. All of the 104 non-survivors experienced tumour recurrence and tumour-related death. Tumour recurrence was found in 133 patients; of these, 101 (75.9%) patients presented with intrahepatic recurrence. Retreatment after recurrence included transarterial chemoembolisation (*n* = 72), surgery (*n* = 4), radiofrequency ablation (*n* = 10), radiotherapy (*n* = 2), sorafenib (*n* = 20), chemotherapy (*n* = 2) and conservative treatment (*n* = 23).

According to their 5-year survival status, patients were divided into survivor (*n* = 97) and non-survivor (*n* = 104) groups. The baseline characteristics of the two groups are summarised in Table 1. AFP, AST, MRI-reported tumour number, MRI-reported tumour size and BCLC stage were found to be significantly different between groups.

**Table 1:** Baseline characteristics of survivor and non-survivor groups.

Characteristics	Survivor (*n* = 97)	Non-survivor (*n* = 104)	*P-*value
Age (years)			0.066
≤60	74 (76.3%)	67 (64.4%)	
>60	23 (23.7%)	37 (35.6%)	
Sex			0.740
Male	81 (83.5%)	85 (81.7%)	
Female	16 (16.5%)	19 (18.3%)	
HBsAg/HCV-ab status			0.724
Negative	15 (15.5%)	18 (17.3%)	
Positive	82 (84.5%)	86 (82.7%)	
Cirrhosis			0.932
Absent	21 (21.6%)	22 (21.2%)	
Present	76 (78.4%)	82 (78.8%)	
Preoperative AFP (ng/mL)			<0.001*
≤7	51 (52.6%)	24 (23.1%)	
>7	46 (47.4%)	80 (76.9%)	
Preoperative ALT (U/L)			0.994
≤40	70 (72.2%)	75 (72.1%)	
>40	27 (27.8%)	29 (27.9%)	
Preoperative AST (U/L)			0.004*
≤45	80 (82.5%)	67 (64.4%)	
>45	17 (17.5%)	37 (35.6%)	
Preoperative TB (μmol/L)			0.429
≤20	79 (81.4%)	89 (85.6%)	
>20	18 (18.6%)	15 (14.4%)	
Preoperative DB (μmol/L)			0.158
≤6	74 (76.3%)	70 (67.3%)	
>6	23 (23.7%)	34 (32.7%)	
Preoperative ALB (g/L)			0.126
<35	4 (4.1%)	10 (9.6%)	
≥35	93 (95.9%)	94 (90.4%)	
Preoperative PLT (10^9^/L)			0.770
<100	18 (18.6%)	21 (20.2%)	
≥100	79 (81.4%)	83 (79.8%)	
Preoperative PT (seconds)			0.573
≤14	89 (91.8%)	93 (89.4%)	
>14	8 (8.2%)	11 (10.6%)	
Preoperative INR			0.261
≤1.5	88 (90.7%)	89 (85.6%)	
>1.5	9 (9.3%)	15 (14.4%)	
Preoperative CP class			1.000
A	96 (99.0%)	103 (99.0%)	
B	1 (1.0%)	1 (1.0%)	
MRI-reported tumour number			0.023*
≤3	94 (96.9%)	92 (88.5%)	
>3	3 (3.1%)	12 (11.5%)	
MRI-reported tumour size (cm)			0.018*
≤2	21 (21.6%)	10 (9.6%)	
>2	76 (78.4%)	94 (90.4%)	
BCLC stage			0.003*
0	19 (19.6%)	8 (7.7%)	
A	72 (74.2%)	76 (73.1%)	
B	6 (6.2%)	20 (19.2%)	

*AFP* alpha-fetoprotein, *ALB* albumin, *ALT* alanine aminotransferase, *AST* aspartate aminotransferase, *BCLC* Barcelona Clinic Liver Cancer, *CP* Child–Pugh, *DB* direct bilirubin, *HBsAg* hepatitis B surface antigen, *HCV-ab* hepatitis C Virus antibody, *INR* international normalised ratio, *PLT* platelet count, *PT* prothrombin time, *TB* total bilirubin, *MRI* magnetic resonance imaging

*P-value < 0.05

Uni- and multivariable logistic regression analyses were performed to determine preoperative clinical risk factors associated with 5-year survival. AFP and AST were found to be independent clinical risk factors in multivariable logistic regression analysis (Table 2).

**Table 2:** Preoperative clinical risk factors for 5-year survival in patients with HCC.

Variable	Univariable analysis		Multivariable analysis	
	OR (95% CI)	*P-*value	OR (95% CI)	*P-*value
Age, >60 years vs. ≤60 years	1.777 (0.959–3.291)	0.068	1.693 (0.849–3.376)	0.135
Sex, female vs. male	1.132 (0.545–2.351)	0.740		
HBsAg/HCV-ab status, positive vs. negative	0.874 (0.413–1.848)	0.724		
Cirrhosis, present vs. absent	1.030 (0.525–2.022)	0.932		
Preoperative AFP level, >7 ng/mL vs. ≤7 ng/mL	3.696 (2.017–6.773)	<0.001*	3.789 (1.986–7.231)	<0.001*
Preoperative ALT level, >40 U/L vs. ≤40 U/L	1.002 (0.541–1.858)	0.994		
Preoperative AST level, >45 U/L vs. ≤45 U/L	2.599 (1.344–5.026)	0.005*	2.692 (1.300–5.573)	0.008*
Preoperative TB level, >20 μmol/L vs. ≤20 μmol/L	0.740 (0.350–1.565)	0.430		
Preoperative DB level, >6 μmol/L vs. ≤6 μmol/L	1.563 (0.839–2.911)	0.159		
Preoperative ALB level, <35 g/L vs. ≥35 g/L	2.473 (0.749–8.166)	0.137		
Preoperative PLT, <100 × 10^9^/L vs. ≥100 × 10^9^/L	1.110 (0.551–2.238)	0.770		
Preoperative PT, >14 s vs. ≤14 s	1.316 (0.506–3.423)	0.574		
Preoperative INR, >1.5 vs. ≤1.5	1.648 (0.685–3.963)	0.264		
Preoperative CP class, B vs. A	0.932 (0.057–15.110)	0.961		
MRI-reported tumour number, >3 vs. ≤3	4.087 (1.117–14.957)	0.033*	1.555 (0.218–11.078)	0.659
MRI-reported tumour size, >2 cm vs. ≤2 cm	2.597 (1.154–5.847)	0.021*	1.959 (0.810–4.739)	0.136
BCLC stage, B vs. 0/A	3.611 (1.384–9.425)	0.009*	2.933 (0.665–12.938)	0.155

*AFP* alpha-fetoprotein, *ALB* albumin, *ALT* alanine aminotransferase, *AST* aspartate aminotransferase, *BCLC* Barcelona Clinic Liver Cancer, *CI* confidence interval, *CP* Child–Pugh, *DB* direct bilirubin, HBsAg hepatitis B surface antigen, *HCC* hepatocellular carcinoma, *HCV-ab* hepatitis C Virus antibody, *INR* international normalised ratio, *PLT* platelet count, *PT* prothrombin time, *TB* total bilirubin, *MRI* magnetic resonance imaging, *OR* odds ratio, vs. versus

*P-value < 0.05

### Feature selection and radiomics signature construction

A total of 80 invalid features were removed, including 16 with infinite values, 60 with null values and 4 with variances of zero. Among the remaining 3064 radiomics features, the 30 most survival-related features were selected. A total of 150 radiomics features were selected for five-fold cross-validation, including 44 from DCEI, 42 from DWI, 29 from T1WI and 35 from T2WI. The distribution of the selected radiomics features is shown in Supplementary Table S1.

**Fig. 3:**
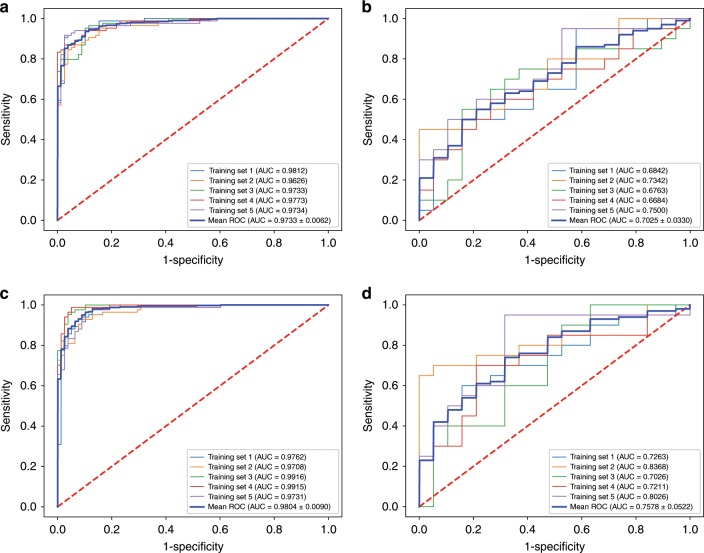
Receiver-operating characteristic (ROC) curves for the radiomics signature and radiomics model. **a** ROC curve for the radiomics signature in the training set, showing a mean area under the curve (AUC) of 0.9733 (95% confidence interval [CI], 0.9671–0.9795). **b** ROC curve for the radiomics signature in the validation set, showing a mean AUC of 0.7025 (95% CI, 0.6695–0.7355). **c** ROC curve for the radiomics model with the addition of preoperative alpha-fetoprotein (AFP) and aspartate aminotransferase (AST) in the training set, showing a mean AUC of 0.9804 (95% CI, 0.9714–0.9894). **d** ROC curve for the radiomics model with the addition of preoperative AFP and AST in the validation set, showing a mean AUC of 0.7578 (95% CI, 0.7056–0.8100).

A radiomics signature was developed based on the selected radiomics features. The setting value for the number of trees in the forest and maximum leaf node was 100 and 5, respectively. The radiomics signature yielded a mean AUC of 0.9733 (95% CI, 0.9671–0.9795) in the training set (Fig. 3a), and 0.7025 (95% CI, 0.6695–0.7355) in the validation set (Fig. 3b).

### Development and performance of radiomics model

**Fig. 4:**
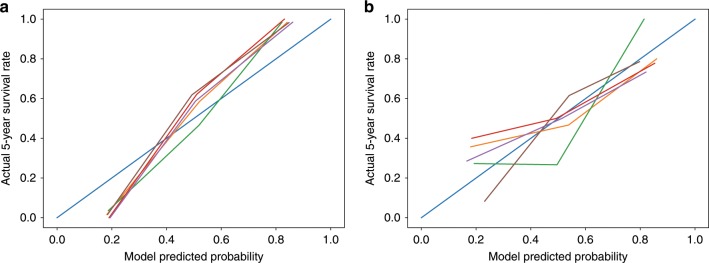
Calibration curves for the radiomics model. Calibration curves for the radiomics model in the training (**a**) and validation (**b**) sets. The diagonal blue line represents the perfect performance of an ideal model. The red, orange, green, purple and brown lines represent the performance of the radiomics model in five different training or validation sets, of which a closer fit to the diagonal blue line indicates a better prediction performance.

Preoperative AFP and AST were integrated into the radiomics model. The list of the selected features and their coefficients are available in Supplementary Tables S2–S6. An individualised radiomics model incorporating the radiomics signature and preoperative AFP and AST showed better discrimination, with a mean AUC of 0.9804 (95% CI, 0.9714–0.9894) in the training set (Fig. 3c), and 0.7578 (95% CI, 0.7056–0.8100) in the validation set (Fig. 3d). The calibration curve demonstrated good agreement between predicted and actual 5-year survival rates in the training (Fig. 4a) and validation (Fig. 4b) sets. The decision curve showed good performance of the radiomics model in terms of clinical application, which added more benefit than either a treat-all or treat-none scheme (Supplementary Fig. S1).

## Discussion

To our knowledge, this is the first MRI-based radiomics model for 5-year survival prediction in patients with HCC. The model integrates the MRI radiomics signature with preoperative AFP and AST, all of which can be easily obtained, to offer prognostic information on long-term survival, which is a key concern for patients. This radiomics model demonstrated satisfactory discriminative ability, and can be used to stratify patients with HCC into risk groups prior to surgery and guide treatment decisions. The non-survivors in our prediction model are regarded as high-risk patients who require close surveillance and may be candidates for clinical trials of perioperative therapies.

Some researchers have developed a number of CT-based radiomics models to predict microscopic vascular invasion, recurrence, and survival in patients with resected HCC.^15–19^ Recently, Guo et al.^25^ found that CT-based radiomics signature, including three texture features, one shape feature and five wavelet features, may enable recurrence prediction for HCC after liver transplantation. With the advantages of multiple parameters, multiple sequences and better soft tissue resolution, MRI shows greater lesion sensitivity and is regarded as the preferred imaging modality for the diagnosis of HCC.^26–28^ In fact, contrast-enhanced MRI is gradually becoming a routine preoperative examination for patients with HCC at many medical centres. Accumulating evidence also suggests the predictive value of an MRI-based radiomics model in nasopharyngeal carcinoma, breast cancer, glioma and cervical cancer.^29–34^ Given the widespread application and predictive value of MRI, it is necessary to build an MRI-based prognosis model for treatment guidance in resectable HCC.

Hui et al.^20^ analysed the largest cross-sectional tumour area on T2WI, DWI and DCEI in 50 patients with HCC and concluded that texture analysis on MRI had the potential to predict early recurrence with up to 84% accuracy using a single parameter. Wu et al.^21^ analysed three-dimensional tumour volume on T1WI and T2WI in 170 patients with HCC and found that the MRI radiomics signature could successfully categorise the grade of HCC; the AUC of the radiomics signature based on T1WI, T2WI and T1WI + T2WI was 0.712, 0.722 and 0.742, respectively. Kim et al.^22^ analysed three-dimensional tumour and peritumoral volume on contrast-enhanced MRI sequences in 167 patients with HCC and developed a radiomics model to predict postoperative recurrence with a c-index value of 0.716. Recently, Zhang et al.^23^ developed a radiomics nomogram based on contrast-enhanced MRI for early postoperative recurrence prediction with an AUC of 0.844. These MRI-based radiomics analyses revealed good predictive ability of MRI in HCC, and suggested that the combination of different MRI sequences may have better prediction performance. Limitations that might weaken the predictive effect of the model in these studies, included small-sample size, single-institution data and single slice- or only a few sequences-derived features.

MRI contains anatomical and functional information: T1WI and T2WI represent tumour features in spatial dimensions, DWI reflects tissue microcirculation, and DCEI is a function of tumour vascularity and cellularity. Therefore, our study took advantage of features extracted from the whole tumour volume on four conventional MRI sequences to maximise detailed tumour characteristics. To the best of our knowledge, this is the only study including four MRI sequences for HCC model building. In this study, the contributions of the different sequences to the prediction model were as follows: DCEI > DWI > T2WI > T1WI. Our results are in good agreement with those reported in previous radiomics studies of HCC, which suggested that the texture parameters on DCEI achieved the best performance compared with DWI or T2WI,^20^ and that the radiomics signature based on T2WI demonstrated better predictive ability compared with T1WI.^21^

Preoperative AFP level is an important prognostic marker of HCC associated with pathological grade, progression and survival. Previous research suggested that patients with HCC and higher serum AFP level may require comprehensive therapy besides surgical resection and close follow-up.^35,36^ Zhou et al.^37^ analysed the prognostic roles of ALT and AST in patients with HCC and B-type hepatitis-associated cirrhosis, and found that only AST was marginally significant in multivariate tests for early recurrence and post-recurrence survival. This study demonstrated significant associations between AFP and AST and 5-year survival, and an obvious improvement was observed when AFP and AST were added to the radiomics model, which is consistent with previous studies.

Much effort was made to optimise the model. Various methods were employed, and the random forest algorithm was selected for feature selection and model construction. The random forest algorithm is a fully non-parametric, machine-learning method, which is highly effective for prediction and variable selection in high-dimensional problems.^38^ Zhang et al.^39^ evaluated the performance of six feature selection methods and nine classification methods, and identified the random forest algorithm as the optimal machine-learning classifier for radiomics-based prediction of failure in advanced nasopharyngeal carcinoma. Akai et al.^15^ found that the combination of radiomics analysis and random forest method may be useful for prognosis prediction in resectable HCC. Five-fold cross-validation was performed for robust assessment. In the process of model building, dividing patients into training and validation groups is common based on the defect of population mismatch, which leads to poor performance in small-sample studies. To address this limitation, we applied five-fold cross-validation to calculate the average result. The combination of random forest and cross-validation methods has been widely used in recent radiomics analyses.^15,40^

There were several limitations in our study. First, the radiomics model was developed based on retrospective data, and the clinical usefulness requires independent validation in further studies. Second, this study collected multicentre MRI data to increase the statistical power and sample size at the expense of increased variability of different scanners; however, we used uniform scanning parameters and MRI signal-intensity normalisation to reduce the possible variability. Third, genomic characteristics were not considered in this study. Radiogenomics is an emerging field exploring the relationships between imaging phenotypes and gene expression.^41^ Some preliminary studies have revealed correlations between phenotypic imaging traits and genomic signatures in patients with HCC.^42,43^ However, the small-sample size and limited imaging features may influence the reliability of the detected image-to-gene associations. Future studies could attempt to incorporate genomic characteristics into the radiomics model to capture more underlying behaviours in HCC. Finally, many other variables could have influenced 5-year survival, such as postoperative pathological characteristics and retreatment after recurrence. As we aimed to develop a preoperative model that could direct clinical trials of perioperative therapies, and only preoperative clinical and radiomics features were included for modelling, it may have limited the predictive effect. However, this preoperative model showed favourable performance, suggesting good predictive value of the preoperative radiomics features for 5-year survival. In the future, we will incorporate other survival-related factors into a postoperative model.

In conclusion, we constructed a convenient and feasible radiomics model that integrated the MRI radiomics signature and preoperative clinical risk factors to predict 5-year survival in patients with resectable HCC. The ability of risk stratification in the preoperative setting can identify patients for clinical trials of perioperative therapies and for additional surveillance after surgery.

## Supplementary information

Supplementary Material

## Data Availability

The data might be made available upon request, and some restrictions will apply.
