# Supplementary material for: MRI-based radiomics model for preoperative prediction of 5-year survival in patients with hepatocellular carcinoma
Source: Br J Cancer. 2020 Jan 15;122(7):978–85. doi: 10.1038/s41416-019-0706-0 (PMC7109104; doi:10.1038/s41416-019-0706-0)
Supplement: Supplementary file 1 — Supplementary Material [file 41416_2019_706_MOESM1_ESM.docx]

**Supplementary Figure S1.**

**
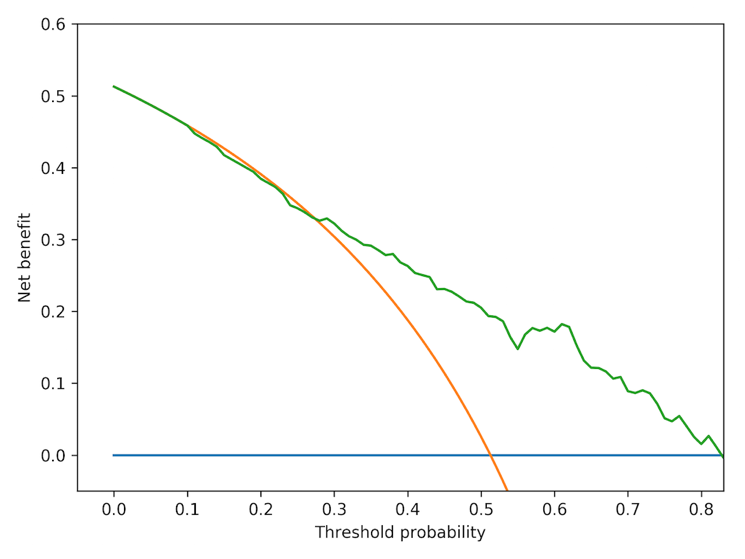
**

**Supplementary Figure legends**

**Figure S1.** Decision curve analysis for the radiomics model. The orange line represents the assumption that all patients are high risk, while the blue line represents the assumption that no patients are high risk. The green line represents the radiomics model. The decision curve shows that if the threshold probability of a patient is in the range of 28% to 83%, the radiomics model adds more benefit than a treat-all or treat-none scheme.

**Supplementary Table S1. Distribution of selected radiomics features in five training sets**

|  | Number of selected features | | | | |
| --- | --- | --- | --- | --- | --- |
| Sequence | Set 1 | Set 2 | Set 3 | Set 4 | Set 5 |
| DCEI | 10 | 6 | 9 | 11 | 8 |
| DWI | 8 | 12 | 10 | 4 | 8 |
| T1WI | 5 | 8 | 4 | 9 | 3 |
| T2WI | 7 | 4 | 7 | 6 | 11 |

Abbreviations: DCEI, dynamic contrast-enhanced imaging; DWI, diffusion weighted imaging; T1WI, T1-weighted imaging; T2WI, T2-weighted imaging

**Supplementary Table S2. Selected features and correlation coefficients in set 1**

| **Selected Features in Set 1** | **Correlation Coefficient** |
| --- | --- |
| [1] DCE_hog_2_0_2_5_3D_Histogram | 0.010023702 |
| [2] DCE_hog_1_0_2_15_3D_Histogram | 0.019587279 |
| [3] DCE_hog_1_0_2_16_3D_Histogram | 0.041581094 |
| [4] DCE_hog_2_0_0_17_3D_Histogram | 0.020896575 |
| [5] DCE_hog_0_0_2_18_3D_Histogram | 0.017125160 |
| [6] DCE_statistic_-1_0_0-Energy | 0.084120340 |
| [7] DCE_statistic_-1_-1_0-SumMean | 0.015313136 |
| [8] DCE_statistic_0_1_-1-Homogeneity | 0.081898052 |
| [9] DCE_statistic_-1_0_-1-MaxProbability | 0.029598826 |
| [10] DCE_statistic_1_0_-1-Homogeneity | 0.033770461 |
| [11] DWI_hog_2_1_2_1_3D_Histogram | 0.013623254 |
| [12] DWI_hog_2_0_0_3_3D_Histogram | 0.024225030 |
| [13] DWI_hog_2_1_0_11_3D_Histogram | 0.048807462 |
| [14] DWI_hog_0_0_1_11_3D_Histogram | 0.016566261 |
| [15] DWI_texture_(32)GLZSM-SmallZoneSizeEmphasis | 0.024604936 |
| [16] DWI_statistic_-1_-1_0-SumMean | 0.035622744 |
| [17] DWI_statistic_1_-1_-1-Entropy | 0.024302138 |
| [18] DWI_statistic_-1_-1_-1-ClusterTendency | 0.036891667 |
| [19] T1_wavelet_LLH-Histogram-Variance | 0.025505112 |
| [20] T1_statistic_-1_1_0-Homogeneity | 0.016443832 |
| [21] T1_statistic_-1_1_0-Variance | 0.047796319 |
| [22] T1_statistic_0_1_-1-Entropy | 0.011133470 |
| [23] T1_statistic_-1_0_-1-lnverse_Variance | 0.033247025 |
| [24] T2_hog_1_1_0_5_3D_Histogram | 0.017289659 |
| [25] T2_hog_2_1_0_9_3D_Histogram | 0.068102110 |
| [26] T2_hog_2_1_0_10_3D_Histogram | 0.026497559 |
| [27] T2_hog_1_2_1_11_3D_Histogram | 0.042586245 |
| [28] T2_hog_2_1_0_17_3D_Histogram | 0.035346395 |
| [29] T2_texture_(2)Histogram-Variance | 0.026705220 |
| [30] T2_wavelet_HHL-Histogram-Entropy | 0.031187945 |
| [31] AFP | 0.030865201 |
| [32] AST | 0.008735795 |

Abbreviations: AFP, alpha-fetoprotein; AST, aspartate aminotransferase

**Supplementary Table S3. Selected features and correlation coefficients in set 2**

| **Selected Features in Set 2** | **Correlation Coefficient** |
| --- | --- |
| [1] DCE_hog_1_0_1_1_3D_Histogram | 0.017684539 |
| [2] DCE_hog_1_0_0_8_3D_Histogram | 0.048571911 |
| [3] DCE_wavelet_LLH-Histogram-Kurtosis | 0.044343969 |
| [4] DCE_statistic_0_1_0-ClusterTendency | 0.036043691 |
| [5] DCE_statistic_1_-1_-1-Homogeneity | 0.028805561 |
| [6] DCE_statistic_-1_-1_-1-Homogeneity | 0.048318194 |
| [7] DWI_hog_1_1_0_4_3D_Histogram | 0.035487711 |
| [8] DWI_hog_2_0_1_4_3D_Histogram | 0.035514326 |
| [9] DWI_hog_2_0_1_6_3D_Histogram | 0.025105598 |
| [10] DWI_hog_1_1_1_10_3D_Histogram | 0.016757359 |
| [11] DWI_hog_0_1_0_16_3D_Histogram | 0.023175114 |
| [12] DWI_hog_2_1_1_17_3D_Histogram | 0.050379643 |
| [13] DWI_hog_0_2_1_18_3D_Histogram | 0.020306027 |
| [14] DWI_wavelet_LHL-Histogram-Energy | 0.088455465 |
| [15] DWI_statistic_-1_0_-1-Inertia | 0.061533539 |
| [16] DWI_statistic_1_0_-1-Homogeneity | 0.023837107 |
| [17] DWI_statistic_1_0_-1-ClusterTendency | 0.007711224 |
| [18] DWI_statistic_1_1_-1-Max Probability | 0.024753509 |
| [19] T1_hog_2_2_1_8_3D_Histogram | 0.011029769 |
| [20] T1_hog_2_2_2_11_3D_Histogram | 0.006250777 |
| [21] T1_hog_0_2_0_14_3D_Histogram | 0.022534178 |
| [22] T1_wavelet_LLL-Histogram-Variance | 0.053715164 |
| [23] T1_statistic_-1_1_-1-MaxProbability | 0.038422045 |
| [24] T1_statistic_1_1_-1-Energy | 0.004916968 |
| [25] T1_statistic_1_-1_-1-Inertia | 0.031881395 |
| [26] T1_statistic_1_1_-1-Homogeneity | 0.049271210 |
| [27] T2_hog_0_0_0_7_3D_Histogram | 0.023034088 |
| [28] T2_texture_(15)GTSDM-Entropy | 0.029752516 |
| [29] T2_statistic_-1_-1_0-Contrast | 0.005261960 |
| [30] T2_statistic_0_-1_-1-ClusterTendency | 0.036176586 |
| [31] AFP | 0.036691372 |
| [32] AST | 0.014277484 |

Abbreviations: AFP, alpha-fetoprotein; AST, aspartate aminotransferase

**Supplementary Table S4. Selected features and correlation coefficients in set 3**

| **Selected Features in Set 3** | **Correlation Coefficient** |
| --- | --- |
| [1] DCE_hog_0_2_1_0_3D_Histogram | 0.011342387 |
| [2] DCE_hog_0_0_0_3_3D_Histogram | 0.025730513 |
| [3] DCE_hog_0_1_2_6_3D_Histogram | 0.025562137 |
| [4] DCE_hog_1_2_2_10_3D_Histogram | 0.011801498 |
| [5] DCE_texture_(33)GLZSM-LargeZoneSizeEmphasis | 0.120142246 |
| [6] DCE_texture_(38)GLZSM-LargeZone/LowGrayEmphasis | 0.062783428 |
| [7] DCE_wavelet_HHL-Histogram-Energy | 0.051257258 |
| [8] DCE_statistic_0_1_0-Inertia | 0.024145557 |
| [9] DCE_statistic_1_1_-1-Inverse Variance | 0.008751133 |
| [10] DWI_hog_0_2_0_3_3D_Histogram | 0.021936050 |
| [11] DWI_hog_2_0_2_7_3D_Histogram | 0.029526103 |
| [12] DWI_hog_1_1_2_8_3D_Histogram | 0.014116827 |
| [13] DWI_hog_0_1_1_11_3D_Histogram | 0.024947570 |
| [14] DWI_hog_1_2_0_19_3D_Histogram | 0.019082543 |
| [15] DWI_wavelet_LHL-Histogram-Variance | 0.052652657 |
| [16] DWI_statistic_0_-1_-1-Contrast | 0.039979313 |
| [17] DWI_statistic_0_-1_-1-Variance | 0.020117429 |
| [18] DWI_statistic_1_0_-1-Contrast | 0.044768014 |
| [19] DWI_statistic_1_1_-1-Inertia | 0.023329236 |
| [20] T1_hog_1_1_2_5_3D_Histogram | 0.011893159 |
| [21] T1_hog_0_2_2_14_3D_Histogram | 0.030722797 |
| [22] T1_hog_0_0_1_16_3D_Histogram | 0.020366487 |
| [23] T1_wavelet_HHH-Histogram-Entropy | 0.042026909 |
| [24] T1_statistic_1_-1_-1-Sum Mean | 0.036574390 |
| [25] T2_hog_2_2_0_10_3D_Histogram | 0.022251549 |
| [26] T2_hog_2_2_1_11_3D_Histogram | 0.018659429 |
| [27] T2_hog_2_0_0_16_3D_Histogram | 0.079553002 |
| [28] T2_hog_2_2_2_16_3D_Histogram | 0.022210293 |
| [29] T2_statistic_0_1_-1-Variance | 0.000000000 |
| [30] T2_statistic_-1_-1_-1-ClusterShade | 0.056595982 |
| [31] AFP | 0.027174102 |
| [32] AST | 0.000000000 |

Abbreviations: AFP, alpha-fetoprotein; AST, aspartate aminotransferase

**Supplementary Table S5. Selected features and correlation coefficients in set 4**

| **Selected Features in Set 4** | **Correlation Coefficient** |
| --- | --- |
| [1] DCE_hog_0_2_0_6_3D_Histogram | 0.019124036 |
| [2] DCE_hog_1_0_0_8_3D_Histogram | 0.035006568 |
| [3] DCE_hog_1_2_2_10_3D_Histogram | 0.003560937 |
| [4] DCE_hog_1_1_1_13_3D_Histogram | 0.025642615 |
| [5] DCE_hog_0_0_1_19_3D_Histogram | 0.009960032 |
| [6] DCE_texture_(33)GLZSM-LargeZoneSizeEmphasis | 0.093201940 |
| [7] DCE_texture_(38)GLZSM-LargeZone/LowGrayEmphasis | 0.035204239 |
| [8] DCE_wavelet_LLL-Histogram-Skewness | 0.035648184 |
| [9] DCE_statistic_0_1_0-MaxProbability | 0.038300113 |
| [10] DCE_statistic_-1_-1_0-Homogeneity | 0.048641847 |
| [11] DCE_statistic_1_0_-1-Homogeneity | 0.066143129 |
| [12] DWI_statistic_0_1_0-Contrast | 0.020634299 |
| [13] DWI_statistic_-1_0_0-Contrast | 0.011082543 |
| [14] DWI_statistic_-1_0_0-Inertia | 0.038341469 |
| [15] DWI_statistic_1_-1_-1-ClusterShade | 0.025959556 |
| [16] T1_hog_0_0_1_7_3D_Histogram | 0.027494946 |
| [17] T1_hog_0_1_1_12_3D_Histogram | 0.022618622 |
| [18] T1_hog_0_1_2_14_3D_Histogram | 0.050632466 |
| [19] T1_wavelet_LLL-Histogram-Skewness | 0.008830211 |
| [20] T1_wavelet_LLL-Histogram-Kurtosis | 0.033258516 |
| [21] T1_wavelet_HLL-Histogram-Entropy | 0.067302671 |
| [22] T1_statistic_-1_-1_-1-Homogeneity | 0.014985744 |
| [23] T1_statistic_-1_1_1_Variance | 0.021104907 |
| [24] T1_statistic_1_1_-1_SumMean | 0.031941987 |
| [25] T2_hog_2_0_2_6_3D_Histogram | 0.034755997 |
| [26] T2_hog_1_2_2_11_3D_Histogram | 0.028346664 |
| [27] T2_hog_2_2_0_12_3D_Histogram | 0.025051901 |
| [28] T2_hog_2_0_1_16_3D_Histogram | 0.063305705 |
| [29] T2_hog_2_2_0_17_3D_Histogram | 0.020613731 |
| [30] T2_statistic_1_1_-1-ClusterShade | 0.022300057 |
| [31] AFP | 0.021004369 |
| [32] AST | 0.000000000 |

Abbreviations: AFP, alpha-fetoprotein; AST, aspartate aminotransferase

**Supplementary Table S6. Selected features and correlation coefficients in set 5**

| **Selected Features in Set 5** | **Correlation Coefficient** |
| --- | --- |
| [1] DCE_hog_2_0_0_1_3D_Histogram | 0.012581740 |
| [2] DCE_hog_2_0_1_1_3D_Histogram | 0.009185468 |
| [3] DCE_hog_0_0_1_6_3D_Histogram | 0.046885439 |
| [4] DCE_hog_0_1_2_13_3D_Histogram | 0.019858967 |
| [5] DCE_statistic_-1_1_0-Homogeneity | 0.053261376 |
| [6] DCE_statistic_0_0_-1-Homogeneity | 0.077884756 |
| [7] DCE_statistic_-1_0_-1-Homogeneity | 0.044011888 |
| [8] DCE_statistic_-1_1_-1-Homogeneity | 0.059329379 |
| [9] DWI_hog_0_1_0_3_3D_Histogram | 0.046783308 |
| [10] DWI_hog_2_0_1_4_3D_Histogram | 0.011955389 |
| [11] DWI_hog_0_1_1_11_3D_Histogram | 0.025678636 |
| [12] DWI_hog_0_2_0_12_3D_Histogram | 0.024323598 |
| [13] DWI_hog_1_1_1_17_3D_Histogram | 0.007692400 |
| [14] DWI_wavelet_HHL-Histogram-Variance | 0.050788892 |
| [15] DWI_statistic_-1_-1_0-Contrast | 0.048316986 |
| [16] DWI_statistic_-1_0_-1-Inertia | 0.020421958 |
| [17] T1_hog_0_0_2_4_3D_Histogram | 0.032693299 |
| [18] T1_wavelet_LHL-Histogram-Kurtosis | 0.086955933 |
| [19] T1_wavelet_HLH-Histogram-Mean | 0.017925671 |
| [20] T2_hog_2_2_1_5_3D_Histogram | 0.019068050 |
| [21] T2_hog_2_1_0_6_3D_Histogram | 0.043789256 |
| [22] T2_hog_0_2_2_6_3D_Histogram | 0.016035142 |
| [23] T2_hog_0_0_1_7_3D_Histogram | 0.001768037 |
| [24] T2_hog_2_0_2_9_3D_Histogram | 0.020532071 |
| [25] T2_hog_1_2_0_16_3D_Histogram | 0.035456221 |
| [26] T2_hog_1_0_2_18_3D_Histogram | 0.012333390 |
| [27] T2_statistic_0_1_0-ClusterTendency | 0.031003121 |
| [28] T2_statistic_-1_-1_0-ClusterShade | 0.028191587 |
| [29] T2_statistic_1_-1_-1-Entropy | 0.015123275 |
| [30] T2_statistic_1_1_-1-Homogeneity | 0.051293545 |
| [31] AFP | 0.024412430 |
| [32] AST | 0.004458793 |

Abbreviations: AFP, alpha-fetoprotein; AST, aspartate aminotransferase
